# The neural correlates of integrated aesthetics between moral and facial beauty

**DOI:** 10.1038/s41598-019-38553-3

**Published:** 2019-02-13

**Authors:** Qiuling Luo, Mengxia Yu, You Li, Lei Mo

**Affiliations:** 1 Institute of Psychology, School of Teacher Education, Zhejiang Normal University, Jinhua, China; 2 Center for the Study of Applied Psychological Application & School of Psychology, South China Normal University, Guangzhou, China; 3 Guangdong Provincial Key Laboratory of Mental Health and Cognitive Science, South China Normal University, Guangzhou, China; 4 Shenzhen Institute of Advanced Technology, Chinese Academy of Sciences, Shenzhen, China

## Abstract

Facial beauty and moral beauty have been suggested to be two significant forms of social aesthetics. However, it remains unknown the extent to which there are neural underpinnings of the integration of these two forms of beauty. In the present study, participants were asked to make general aesthetic judgments of facial portraits and moral descriptions while collecting fMRI data. The facial portrait and moral description were randomly paired. Neurally, the appreciation of facial beauty and moral beauty recruited a common network involving the middle occipital gyrus (MOG) and medial orbitofrontal cortex (mOFC). The activities of the mOFC varied across aesthetic conditions, while the MOG was specifically activated in the most beautiful condition. In addition, there was a bilateral insular cortex response to ugliness specifically in the congruent aesthetic conditions, while SMA was selectively responsive to the most ugly condition. Activity associated with aesthetic conflict between facial and moral aesthetic information was limited to the medial prefrontal cortex (mPFC), with enhanced response to the incongruent condition compared to the congruent condition. These findings provide novel neural evidence for the integrated aesthetics of social beauty and suggest that integrated aesthetics is a more complex cognitive process than aesthetics restricted to a single modality.

## Introduction

Facial beauty and moral beauty have been recognized as two significant forms of social information in human communication. Facial beauty, which refers to typical visual attractiveness, is based on the combination of sensory and symbolic elements^1,2^. Physical features such as averageness and symmetry are consistently identified as contributors to facial attractiveness^3^. By contrast, moral beauty, which refers to a complex, physically independent beauty, involves abstract social meanings^4–7^. Moral beauty is the expression of humanity, virtue and talents, and is based on the understanding of social norms^8–10^. Though facial and moral aesthetics present and are characterized independently, they interact with each other. On one hand, the facial aesthetic influences moral judgment, representing the Beauty-is-Good stereotype^11,12^. On the other hand, the moral aesthetic influences the viewer’s perception of target faces, referring to the Good-is-Beauty stereotype^13–16^.

Recently, increasing attention has been directed to the neural mechanisms of social aesthetics. Research on neuroaesthetics has shown that social beauty recruits several specific neural regions that are commonly involved in facial and moral aesthetics. The first candidate is the mOFC. The mOFC has been suggested to be the center for facial aesthetics^17–20^. Compared to unattractive faces, attractive faces have been evidenced to elicit greater activity in the mOFC area^19,21^. The mOFC has also been shown to be recruited in moral aesthetics. Moral beauty (e.g. altruistic behaviour), compared to the morally neutral stimulus, was found significantly activated the mOFC in addition to other advanced cerebral regions^10,22,23^. Additionally, evidence from research on brain damage indicated that OFC lesions result in poor practical judgments^24^ and impaired moral behavior^25^, which provides further evidence of the role of mOFC in moral processing^26^.

There is also evidence suggesting that the insular cortex may be recruited in facial and moral aesthetics^27–29^. Specifically, the activity of this region is enhanced when responding to unattractive faces^21,28,29^ as well as for negative moralities^27,28^. In sum, these studies lead to a consistent conclusion that facial and moral beauties recruit a common network involving the mOFC and insular cortex and suggest that the overlap of neural mechanisms for facial and moral beauty may explain their interaction in the complex processing of social aesthetics.

Although previous neuroimaging research has greatly enhanced our understanding of responses to facial and moral beauty, previous work has not addressed the processing of both of these types of beauty in the same study, leaving questions about the integration of this information completely unanswered. Facial appearance was efficient to evoke trait inference implicitly^11,30,31^. It has been suggested that specific features from a face drive these trait judgments^32^. Also personal traits knowledge could shape processing of the target face^11^. Specifically, one of our experiments showed that even a short exposure to traits information modulated the neural responses to the target faces^33^. These findings have thus suggested an interactive relationship between facial and trait evaluations. However, information of facial or moral beauty was solely manipulated in these studies; it is therefore unclear whether the values of these two forms of beauty are coded along a single dimension and could be integrated into an entirety. In a recent study of Zhang, participants were firstly asked to rate the attractiveness of a set of facial stimuli with varying attractiveness. After two weeks, they performed the attractiveness ratings of the same faces which were presented along with positive, negative, or no personality information. It was found that positive traits promoted facial attractiveness, whereas negative traits reduced facial attractiveness^34^, suggesting a considerable contribution of personality traits to the evaluation of facial attractiveness. Though information of facial and moral beauty was manipulated in this study, they were weighted unevenly due to experimental settings. In the reality, however, facial beauty and moral beauty are always simultaneously involved in human beings. Hence, it would be of high ecological validity to examine how these two forms of information are processed and integrated naturally, which may also provide a window to understand integration of aesthetics from different modalities. The present research was designed to fill this important gap in the literature by examining the neural basis of the integrated evaluation of facial and moral aesthetics. To do so, we addressed the following questions: Are the mOFC and insular cortex involved in integrated aesthetics as in aesthetics that is restricted to a single modality? Are there any regions that are selectively involved in aesthetic conflict?

In the current study, functional MRI was adopted to assess neural activity while participants made general aesthetic judgments of the target in four independent conditions: facial ugliness-moral ugliness (FUMU), facial ugliness-moral beauty (FUMB), facial beauty-moral ugliness (FBMU), and facial beauty-moral beauty (FBMB). A filler condition with neutral aesthetic information was included in the experiment. We anticipated that we would identify neural correlates in three types of network: the network of beauty, the network of ugliness, and the network of aesthetic conflict. The network of beauty in this research was identified by the conjunction of facial beauty and moral beauty, whereas the network of ugliness was identified by the conjunction of facial and moral ugliness. Based on previous findings, the activity of the mOFC was expected to be associated with both facial and moral beauty in our study, and activity of the insula may be elicited by both facial and moral ugliness. Finally, the network of aesthetic conflict was investigated by examining the contrast of conflicted vs. un-conflicted conditions. As the mPFC was sensitive to cognitive or emotional conflict^29^, we hypothesized that this area would be involved in aesthetic conflict rather than aesthetic non-conflict in the present study.

## Materials and Methods

### Participants

Twenty-four right-handed, undergraduate students with no history of neurological or psychiatric problems were recruited as participants. Only females were included to eliminate possible confusion between the gender of the participants and the gender of the stimuli. More importantly, females are suggested to have more consistent aesthetic ratings across participants on heterosexual faces than males^35^. The data from three participants were omitted from the analyses because of the ghost image resulting from machine problem, which resulted in a total of 21 participants (M age = 21.619 years, SD = 1.627) for final analyses. Additionally, the behavioral data for one experimental run for one participant were excluded from analyses due to a hard disk malfunction. All participants were assessed before scan to ensure normal or corrected-to-normal vision. After receiving a complete description of the study, participants gave written informed consent. The study was approved by the ethical review board of the Department of Psychology, South China Normal University. All methods used in the current study were performed in accordance with the relevant guidelines and regulations of the ethical review board.

### Stimuli

Two types of stimuli were used in the present study: facial portraits, which represented facial beauty, and moral descriptions, which represented moral beauty. Given the variation of aesthetic appreciation for stimuli across participants (i.e. one participant might see a stimulus as beautiful, while another might see it as ugly), a specific material set was created for each individual participant.

#### Facial Portraits

Four hundred male Asian photographs were selected mainly from the Native Chinese Facial Affective Picture System^36^ and databases of South China Normal University^10^. Photos from the Internet were also included in order to have enough highly attractive faces. All photos were taken from a frontal view, and all photo subjects were non-famous, had a forward eye-gaze, and had a neutral expression. All photos were digitized in grey scale, and spatially downsized and cropped to remove hair and ears, leaving only a facial mask. After that, all photographs were converted into the dimensions of 187 × 216 pixels on a black background. One month before the study, a pilot study was conducted on 68 participants in order to build individual stimuli sets including more attractive faces, medium attractive faces, and less attractive faces. All volunteers were asked to evaluate the aesthetics of the facial stimuli on a 7-point scale (1 = extremely ugly, 7 = extremely beautiful), yielding 96 most beautiful faces, 96 least beautiful faces, and 48 medium beautiful faces for each participant.

#### Moral Descriptions

Four hundred short sentences were created to describe hypothetical past actions that conveyed different levels of moral beauty with the same requirements but with a larger sample compared to our previous work^33^. In the pilot study, the same 68 participants evaluated the aesthetic of these 400 candidate descriptions on a 7-point scale (1 = extremely ugly, 7 = extremely beautiful), forming three stimulus sets (96 moral beauty descriptions, 48 moral neutral, and 96 moral ugliness descriptions) for each participant. For instance, “He saved a drowning child” represents the moral beauty category; “He chatted with classmates” represents the moral neutral category; and “He betrayed friends for money” represents the moral ugliness category.

The selected facial and descriptive stimuli were randomly paired for each of the pilot study participants who also participated in the formal experiment, generating integrated aesthetic stimuli that fit the following five conditions with an equal number in each: Facial Ugly-Moral Ugly (FUMU), Facial Ugly-Moral Beauty (FUMB), Facial Beauty-Moral Ugly (FBMU), Facial Beauty-Moral Beauty (FBMB) experimental conditions, and Facial Neutral-Moral Neutral (Neutral) control condition. Twenty-four of the 68 pilot participants took part in the experiment.

### Procedure

Three consecutive phases were conducted during the experiment: practicing, fMRI scanning, and post-scan rating. The practice phase implemented the same procedure as the scan phase but with different stimuli in order to familiarize participants with the process. During the scanning, an event related (ER) design was applied with three runs of 80 trials each. Each run contained an equal number of trials representing each of the five conditions (16 each). Every run started with a fixation cross lasting for 6 seconds, followed by the experimental trials presented in a random order.

In a single scan trial, an attractive, neutral, or unattractive face was presented at the center of the screen along with a description of either a positive, neutral, or negative behavior below for 4000 ms. This was followed by the evaluation interface where participants appraised the general aesthetic of the target on a 4-point scale as quickly as possible within 2000 ms by considering the facial and moral attractiveness. Using a response box with buttons labeled 1 to 4, half of the participants made a left index-finger response (i.e., 1, 2) to beautiful stimuli and a right index-finger response (i.e., 3, 4) to ugly stimuli. Reverse responses were used for the other half of participants to balance any influence of movement on data. Once the response was given, the rating bar disappeared and was followed by a blank display for a random period between 500–5000 ms. The scanning phase lasted approximately 40 minutes. The post rating was done in an independent testing room immediately after the fMRI scan to examine the aesthetic stability and the efficiency of stimuli selection. Participants were asked to perform the aesthetic judgment of the facial stimuli and descriptive stimuli on a 4-point scale. All the stimuli and the rating scale were the same as those applied in fMRI scanning for each participant.

### fMRI data acquisition and processing

The fMRI data were acquired on a 3 T Siemens Trio scanner with a 12-channel phase array head coil. Functional images were obtained with a T2-weighted gradient echo planner imaging sequence (3.5 mm thickness; 0.7 mm gap; 64 × 64 matrix; 34 slices; TE = 30 ms; TR = 2000 ms; flip angle = 90°; FOV = 192 mm) parallel to the AP-PC plane, bottom to top interleaved. There were three runs for the whole test, attaining 315 volumes for a single run.

The preprocessing and statistical analyses for all images were conducted by SPM8 (Welcome Trust Center for Imaging, London, UK; http://www.fil.ion.ucl.ac.uk/spm). In preprocessing, after discarding the first three volumes, images were spatially realigned to the first volume to correct for head movements. All individual runs had <3 mm maximum displacement or <1° rotation. These aligned functional images were then spatially normalized into the Montreal Neurological Institute (MNI) template and resampled with voxel size of 2 × 2 × 2 mm^3^. After that, the data were smoothed with an isotropic full-width half-maximum 8 mm Gaussian kernel.

At the individual level, a general linear model was applied to the fMRI time series where stimulus onset was modeled as a single impulse response function and convolved with canonical hemodynamic response function (HRF). Five conditions (FUMU, FUMB, FBMU, FBMB, and Neutral) were modeled as regressors of interest while six head motions were modeled as covariates of no interest. A high-pass filter with a cut-off period of 128 s was used to remove any low-frequency noise. Subsequently, the contrast images were entered into the group level analysis with participants as a random factor.

First, to identify the special cortical network of beauty during integrated aesthetics, we conducted the contrast of facial beauty “(FBMB + FBMU) − (FUMB + FUMU)” and moral beauty “(FBMB + FUMB) − (FBMU + FUMU)” via the flexible factorial analysis in SPM8. We then computed a conjunction between these two contrasts to achieve common cortical regions involved in facial and moral beauty representation. Secondly, a similar procedure was applied in the analyses of the common cerebral representation of ugliness, but contrary to expectation no cortical areas were identified. It is possible that the neural substrates of ugliness during integrated aesthetics do not simply involve facial ugliness and moral ugliness. The inclusion of aesthetic conflict may have an impact on this procedure. In other words, only the ugliness under the congruent condition would be processed, FUMU > FBMB. Consequently, an additional analysis was performed to examine this conjecture. We computed the contrast “4FUMU − 1FUMB − 1FBMU − 1FBMB” to explore the specific areas that were sensitive to the congruent condition where face and morality were both ugly. Thirdly, in the present study, the incongruency between facial and moral aesthetic information leads to a particular condition, aesthetic conflict, which was identified by the contrast of “(FUMB + FBMU) − (FBMB + FUMU)”. All analyses were conducted at a voxel threshold of *p* < 0.001 (uncorrected) and a cluster threshold of *p* < 0.05 (FDR corrected). The extracted imaging data were converted into Z scores for ANOVAs.

## Results

### The efficiency of stimuli selection

In the present study, aesthetic stimuli were selected based on the pilot study and re-evaluated after fMRI scanning. To examine the efficiency of the aesthetic stimuli selection, a repeated measures ANOVA was conducted among beautiful, neutral, and ugly stimuli on the post-scan aesthetic rating respectively for facial and moral stimuli sets. In this analysis, aesthetic ratings for half of the participants were converted into the same standard as the other half of the participants (i.e., reverse coded), with larger numbers representing higher beauty. The response time data were not included in the session because there was no time limitation for the stimuli evaluation.

With regard to facial stimuli, a significant difference in rating was found, *F* (2, 40) = 634.11, *p* < 0.001, ${{\rm{\eta }}}_{{\rm{p}}}^{2}$ = 0.969. The aesthetic ratings decreased from beautiful (3.401 ± 0.299) to neutral (2.304 ± 0.345), then to ugly faces (1.347 ± 0.242), with ratings significantly differing between each kind of face, *ps* < 0.001. Significant differences among the three kinds of moral descriptions were also found, *F* (2, 40) = 519.59, *p* < 0.001, ${{\rm{\eta }}}_{{\rm{p}}}^{2}$ = 0.963, with the largest aesthetic rating for moral beauty (3.554 ± 0.240), the smallest aesthetic rating for moral ugliness (1.385 ± 0.193), and a medium rating for morally neutral (2.833 ± 0.259). Further analyses indicated that the aesthetic ratings significantly differed between each kind of moral description, *ps* < 0.001.

### Integrated aesthetic evaluation

**Figure 1:**
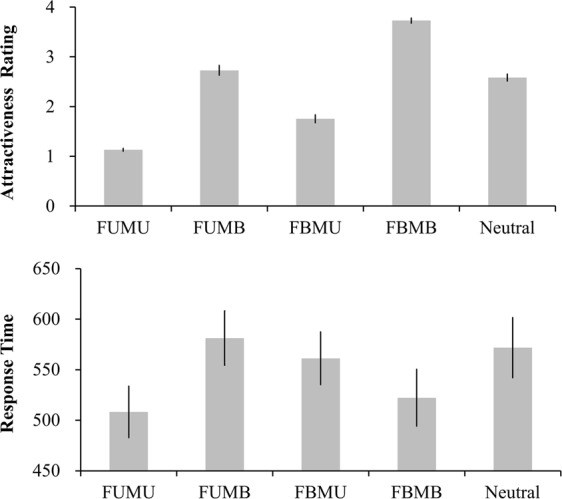
Results of aesthetic rating and response time.

The upper panel of Fig. 1 shows the aesthetic ratings for all conditions. A repeated measures ANOVA with condition as the independent variable resulted in a significant main effect, *F* (4, 80) = 225.590, *p* = 0.000, ${{\rm{\eta }}}_{{\rm{p}}}^{2}$ = 0.919. The FBMB condition had the highest aesthetic ratings, whereas the FUMU condition had the lowest aesthetic ratings. The other three conditions had medium ratings which were significantly smaller than the those in FBMB condition and significantly larger than those in FUMU condition, *ps* < 0.001. Moreover, FBMU was rated significantly less beautiful than FUMB and Neutral, *ps* < 0.001, while FUMB was rated slightly more beautiful than Neutral, *p* = 0.066.

The response times during integrated aesthetics were also significantly different among conditions, *F* (4, 80) = 8.565, *p* = 0.000, ${{\rm{\eta }}}_{{\rm{p}}}^{2}$ = 0.300, please see the lower panel of Fig. 1. Further analyses showed that participants took the shortest time to evaluate in the FBMB and FUMU conditions, significantly shorter than in the other conditions, *ps* < 0.03. The response times in all five conditions were as follows: FUMU: 508 ± 116 ms; FBMB: 522 ± 129 ms; FBMU: 561 ± 120 ms; FUMB: 581 ± 122 ms; and Neutral: 572 ± 137 ms.

### Imaging results

#### Neural networks of beauty during integrated aesthetics

Unlike simple aesthetics, integrated aesthetics present two kinds of aesthetic information about a target; therefore, the neural networks of beauty during integrated aesthetics were supposed to be involved both in facial and moral beauty. The mutual networks of facial beauty and moral beauty were calculated via the following three steps.

First, we identified the neural network involved in facial beauty indexed by the contrast of “(FBMB + FBMU) − (FUMB + FUMU).” Compared to facial ugliness, facial beauty significantly activated the bilateral middle occipital gyrus (MOG) and mOFC. Second, the neural network of moral beauty, reflected in the contrast of “(FBMB + FUMB) − (FBMU + FUMU),” was localized in the lingual gyrus and mOFC. Moreover, neural activity in the frontal regions including the superior frontal gyrus (SFG) and inferior frontal gyrus (IFG) was also found (see Table 1).

**Table 1:** Brain regions showing significant increased BOLD response to beauty, ugliness, and conflict during integrated aesthetics.

Brain regions	R/L	x	y	z	T	Z	Ke	BA
***Face beauty***								
MOG	R	50	−74	0	9.15	7.55	12213	18,19
MOG	L	−44	−84	0	5.17	4.78	579	19
mOFC		−4	38	−22	4.76	4.45	881	11
***Moral beauty***								
SFG	R	20	42	40	6.69	5.95	464	9
IFG	R	46	42	8	6.22	5.60	559	46
Calcarines/Lingual gyrus	R	16	−92	0	5.35	4.93	1083	17
mOFC		6	68	4	4.79	4.48	234	11
***Conjunction of face beauty and moral beauty***								
MOG	R	10	−90	0	4.61	4.33	296	17,18
mOFC (SVC)		6	62	−12	4.27	4.04	90	11
***Face ugly***								
Cuneus	L	−16	−98	−4	5.53	5.07	244	17
Middle frontal gyrus	L	−46	42	24	5.31	4.90	370	46
IPL/Supramarginal	L	−64	−36	40	5.14	4.77	269	40
***Moral ugly***								
Medial SFG		−4	50	26	7.02	6.18	1027	9,10
MTG	R	60	−10	−10	6.93	6.11	476	21
MTG	L	−60	−10	−10	6.66	5.92	1193	21
Uvula	R	22	−76	−34	6.35	5.70	507	
TPJ	L	−58	−52	28	6.33	5.68	1388	40
Superior temporal pole	L	−44	4	−36	5.62	5.15	1947	21,38
SFG		−10	20	60	4.71	4.41	251	6
Insular(SVC)	R	44	−4	18	3.47	3.34	6	13
***Conjunction of face ugly and moral ugly***								
None								
***Largest response in FUMU***								
Insular	R	36	6	14	6.58	5.86	1230	13
Insular	L	−36	4	−6	5.62	5.14	1454	13
Fusiform	L	−34	−42	−22	6.40	5.73	3510	37
TPJ	R	60	−28	28	5.42	4.99	1031	40,42
Cuneus	L	−12	−86	28	5.23	4.84	375	19,18
Precuneus/cingulate		−10	−42	46	4.75	4.45	285	7
SMA/PCC		4	−16	50	4.61	4.33	593	31,6
***Aesthetic conflict***								
Media frontal gyrus		10	36	48	9.88	Inf	18229	8,9,10
IPL, AG	R	56	−62	42	8.89	7.39	2279	40
IPL/SMG	L	−34	−48	38	6.99	6.16	1189	40
ITG	R	66	−22	−22	5.65	5.17	305	20,21
Precuneus		12	−72	50	5.51	5.06	695	7
Cerebellum	L	−26	−74	−32	4.91	4.58	490	

MNI coordinate: MOG: middle occipital gyrus; SFG: superior frontal gyrus; IFG: inferior frontal gyrus; IPL: inferior parietal lobe; MTG: middle temporal gyrus; ITG: inferior temporal gyrus; STG: superior temporal gyrus; SPL: superior parietal lobe. R, Right; L, Left; BA: Broadmann area.

**Figure 2:**
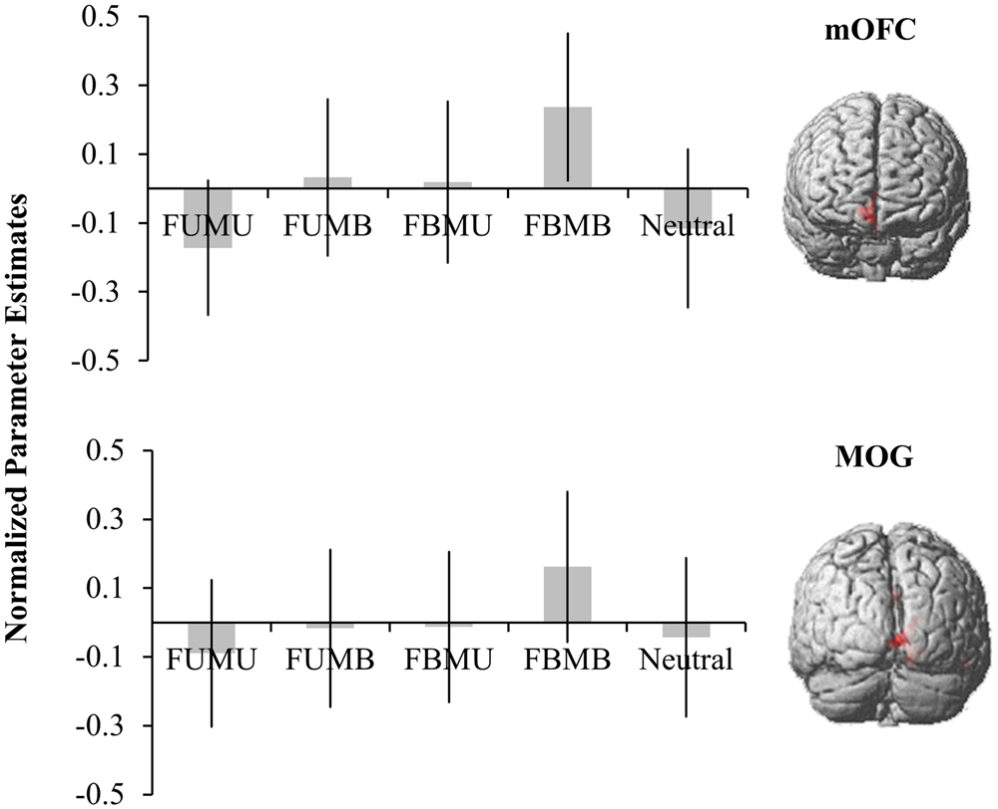
The neural networks commonly modulated by facial beauty and moral beauty.

Third, the conjunction between the facial beauty and moral beauty contrasts was further calculated in order to localize the mutual neural network for aesthetics. It was found that the MOG and mOFC were activated both in the appreciations of facial beauty and moral beauty. As can be seen in Fig. 2, these two regions showed a similar activation pattern. The activity of the mOFC was highest in the FBMB condition and lowest in the FUMU condition. The activity of the mOFC in FBMB was significantly larger than in the other three conditions, *ps* < 0.025, and marginally significantly larger than FBMU, *p* = 0.058. The activity of the mOFC in FUMU was significantly smaller than the other three conditions except the neutral condition, *p* = 0.024, and it was marginally significantly smaller than FBMU, *p* = 0.062. The MOG specifically responded to pure beauty, with the largest activation in FBMB and but lower activation in the other four conditions (with the other four conditions being comparable)*, ps* < 0.001.

#### Neural networks of ugliness during integrated aesthetics

The specific neural network of ugliness was dissociated from that of beauty. The cuneus, left middle frontal gyrus (MFG), and left inferior parietal lobe (IPL) were activated by facial ugliness compared to facial beauty; whereas different neural regions including superior frontal gyrus (SFG), bilateral middle temporal gyrus (MTG), right uvula, left superior temporal lobe, and left TPJ showed increased activity in response to moral ugliness compared to moral beauty. No common region was identified in the processing of both facial and moral ugliness (see Table 1). This result seems to contradict previous findings, which found common areas such as the insula activated in response to decreased attractiveness and goodness^35^.

We suppose that this contradiction could be ascribed to how the special aesthetics context in the present study differed from the previous simple aesthetics context. In our study, the combination of two kinds of beauty information provided a unique opportunity to specify the role of insula during complex aesthetics. If the insula activation is associated with the presence of ugliness/badness or the absence of attractiveness/goodness or both? The absence of significant activation in insular cortex for both facial and moral ugliness leads to a more likely possibility that the insula may only be sensitive to pure ugliness, which means that the activation of this area could be suppressed by any incongruently aesthetic information. To test this hypothesis, the specific region response to pure ugliness (i.e., FUMU) was calculated, resulting in the bilateral insula, right TPJ, and SMA being activated significantly more in the FUMU condition than in other conditions.

**Figure 3:**
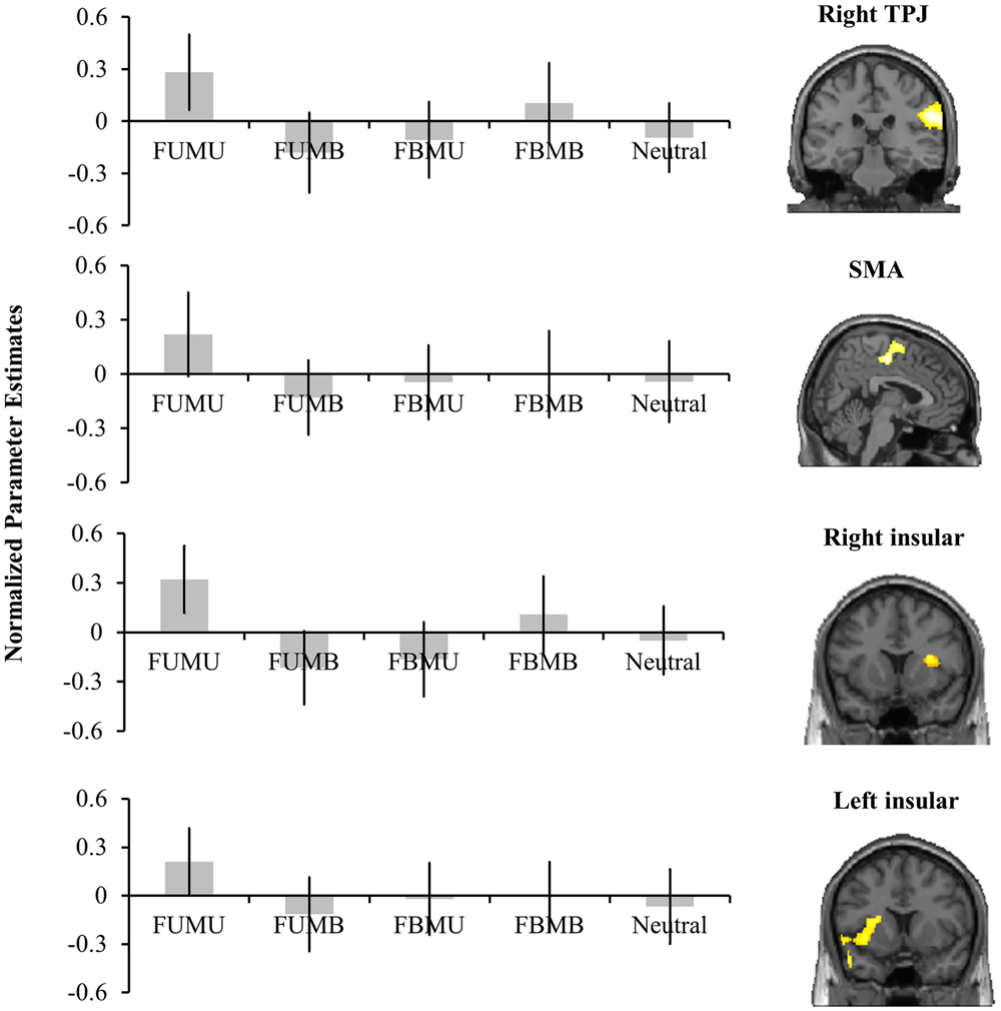
The specific regions responded most to FUMU.

The activation of right insular in the FUMB, FBMU, and Neutral conditions were even significantly smaller than that of the FBMB condition, *ps* < 0.02. Moreover, the activity of this area was also significantly smaller in FUMB than in the Neutral condition, *p* = 0.018. Though in the left insular some statistically significant differences among FUMB, FBMU, FBMB, and Neutral vanished, we still found that the activation in FUMB was significantly smaller than that in the FBMB condition, *p* = 0.031. The TPJ showed the largest response in the FUMU condition, followed by the FBMB, and the lowest response in the other three conditions. By contrast, SMA showed the largest response in the FUMU condition and comparable smaller responses in the other four conditions. Most importantly, the activation of insular cortex was also observed. As can be seen in Fig. 3, the bilateral insular cortex showed a similar activation pattern, with the largest response in the FUMU condition, significantly larger than that in the other conditions, *ps* < 0.001; the smallest response was seen in the FUMB, FBMU, and Neutral conditions.

#### Neural networks for aesthetic conflict

**Figure 4:**
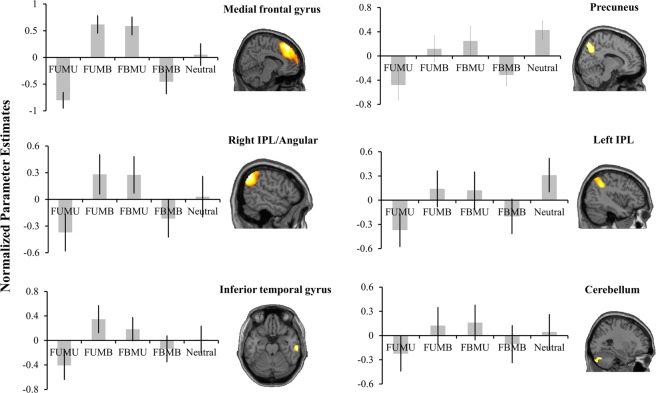
Brain regions’ response to aesthetic conflict.

A special circumstance during integrated aesthetics is aesthetic conflict that contains beautiful and ugly information at the same time, such as the FUMB and FBMU conditions. The contrast of (FUMB + FBMU) − (FBMB + FUMU) revealed significant activations of mPFC, bilateral IPL, right ITG, and left cerebellum for aesthetic conflict compared to aesthetic non-conflict (see Table 1 and Fig. 4).

**Figure 5:**
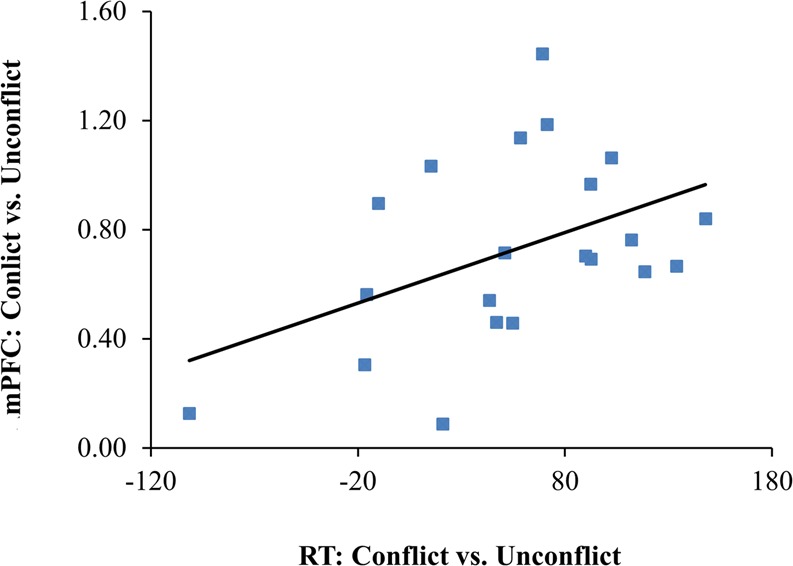
The scatter diagram of the mPFC and the response time difference between conflicted and un-conflicted conditions.

To further examine the relationship between the activation of these brain regions and aesthetic conflict, a Pearson correlation analysis was performed on the region activation difference and response time difference between conflicted vs. un-conflicted conditions. It was found that only mPFC was linearly positively correlated with aesthetic conflict, *r* = 0.448, *p* = 0.042, please see Fig. 5.

## Discussion

The present research investigated the neural mechanisms underlying the integrated aesthetics of facial beauty and moral beauty. Behavioral findings indicated that the integrated aesthetic process was mutually influenced by both facial and moral beauty information, as indexed by response time and aesthetic ratings. Intriguingly, neuroimaging results suggested three main findings about the neural correlates of integrated aesthetics: First, the neural representation of beauty was localized in the mOFC and MOG. The activity of the mOFC was collectively modulated by facial and moral beauty, while the MOG showed a selective response to the most beautiful condition. Second, none of the brain regions was commonly involved in the processing of both facial ugliness and moral ugliness. However, the insula was found to be activated for ugliness especially in un-conflicted conditions. Third, the dmPFC was recruited in aesthetic conflict. Increased activity was associated with incongruency between facial and moral aesthetic information.

The mOFC has commonly been suggested as an important reward related region^37,38^ involved in the processing of different kinds of positive information, such as information about artistic work, delicious food, pleasant odors, harmonious music, and socially rewarded money^39^. In recent decades, the mOFC was introduced to the research on the aesthetic domain when it was shown to demonstrate a consistent sensitivity to facial attractiveness^19,21,40^. The mOFC has also been shown to be involved in the processing of abstract moral beauty or moral goodness^10,24,26^. Further, Tsukiura and his colleagues replicated the activation of the mOFC in facial aesthetic and moral aesthetic judgments. It was found that the mOFC was associated with both facial and moral beauty, with activity being positively and linearly correlated with the degree of beauty^41^.

In accordance with previous studies, the mOFC in the present study showed pronounced response to facial and moral beauty. Furthermore, the activation of the mOFC varied among the experimental conditions. Facial and moral stimuli that included beauty information during integrated aesthetic judgments increased the activity of the mOFC, while any non-beauty (ugliness or neutral) information present in the facial or moral stimuli reduced the activities of mOFC during integrated aesthetics. Therefore, these findings indicate that the mOFC is the integrated center for processing social beauty, and they make a significant contribution to research on aesthetics by advancing our current knowledge of the mOFC and beauty appreciation. It is worth noting that no reward subcortices, such as caudate, nucleus accumbens or putamen, were activated during integrated aesthetics, which may due to the inclusion of advanced moral beauty^10^ (Wang *et al*., 2014) or the ER design^17,20^ in the present study.

Besides these reward related brain regions, the MOG as a core visual processing center was found to be significantly activated in the pure beauty condition, which may reflect a specific sufficient visual processing for facially and morally beautiful individuals^42–44^. The current findings are in line with previous research that reported a positive relationship between aesthetic judgment and activation of the occipital region and its adjacent regions^45,46^. However, an inverse activated pattern in the MOG was reported in other research^47^. It is likely that task demands and contextual circumstances modulated aesthetic processing in the MOG region, contributing to inconsistencies with the results of previous studies^48^. Given that the explanation of aesthetic processing in terms of MOG activation is still controversial, future research is needed to disentangle this issue.

The appreciation of ugliness is another crucial element in aesthetics^48^. The insula, which is the center of negative stimulus processing^49,50^ has also been identified as the key to ugliness processing in numerous previous studies^51^. The current study further found that the bilateral insular cortex was largely activated in the FUMU pure ugliness condition and to a lesser degree in FBMB. Intriguingly, the activity of the insular cortex during incongruent aesthetics was suppressed, with an even smaller response than to the FBMB condition. Moreover, the activations of the insular cortex were not found either for facial ugliness or moral ugliness during integrated aesthetics. These findings seem to contradict previous research; however, they shed light on our understanding of the insular cortex and its relationship to ugliness appreciation. First, from the perspective of neural networks, the appreciation of beauty and ugliness are two independent systems rather than part of an aesthetic continuum. The mOFC takes part in the evaluation of beauty and not-beauty, while the insula is involved in the evaluation of ugliness and not-ugliness. Second, the insula was sensitive to pure ugliness. Stimuli presenting beauty and ugliness simultaneously would be evaluated in the mOFC instead of in the insular cortex. The second finding was specifically significant for clarifying the function of the insular cortex in aesthetics, a big step in the development of neuroaesthetics.

Another finding of the present study concerns the neural correlates of aesthetic conflict. The mPFC was the only region involved in this process, with a linear correlation between the degree of conflict. The involvement of the mPFC is in agreement with previous findings. The mPFC has been commonly deemed as the center of conflict processing. Moreover, Kevin and his colleagues further supposed that mPFC was involved in emotional conflict, but also cognitive conflict^52,53^. Given that, the activation of the mPFC in the current study may imply that complex processes of emotional and cognitive conflict were involved in integrated aesthetics when incongruent information was present.

Briefly, the present study adopted fMRI technique to examine the neural correlates of social aesthetic integration. Our findings suggest commonalities and discriminative processes involved in integrated aesthetics compared to simple aesthetics, which would contribute to the understanding of the essence of social beauty and social aesthetics. However, there are still some limitations. First, the use of an explicit task in the present study may introduce the influence of attention and intention. Second, only female participants and male stimuli were included in our study, and therefore the gender effect during integrated aesthetics could not be tested in our research.

## Conclusion

Taken together, a neural activation model about integrated aesthetics can be proposed. During integrated aesthetics, independent networks were recruited according to the valence and consistency of stimuli information. Once congruent aesthetic stimuli were shown, e.g., FBMB and FUMU, the mOFC was responsible for the evaluation of beauty and the insular cortex was responsible for the evaluation of ugliness. The activity of both areas varied according to the level of beauty or ugliness. Once incongruent aesthetic stimuli were present, e.g., FBMU and FUMB, the mOFC still responded to beauty while the activation of the insular cortex was suppressed. Moreover, the aesthetic conflict between facial and moral information in this situation was processed in the mPFC.

## Data Availability

The data of current study can be obtained by emailing the corresponding authors.
